# Treatment Regret in Patients Undergoing Minimally Invasive Treatments for Benign Prostatic Hyperplasia

**DOI:** 10.3390/jcm15072807

**Published:** 2026-04-07

**Authors:** Riccardo Lombardo, Antonio Luigi Pastore, Beatrice Turchi, Antonio Franco, Matteo Romagnoli, Yazan Al Salhi, Andrea Fuschi, Cristian Fiori, Silvia Secco, Sabrina De Cillis, Alberto Olivero, Antonio Nacchia, Antonio Cicione, Luca Cindolo, Giorgia Tema, Andrea Tubaro, Cosimo De Nunzio

**Affiliations:** 1 Department of Urology, Sapienza University of Rome, 00189 Rome, Italy; antopast@hotmail.com (A.L.P.); beatrice.turchi@uniroma1.it (B.T.); antonio.franco@uniroma1.it (A.F.); matteo.romagnoli@uniroma1.it (M.R.); yazan.alsalhi@uniroma1.it (Y.A.S.); andrea.fuschi@uniroma1.it (A.F.); sabrinatitti.decillis@gmail.com (S.D.C.); antonio.nacchia@uniroma1.it (A.N.); antonio.cicione@uniroma1.it (A.C.); giorgiat88@hotmail.com (G.T.); andrea.tubaro@hotmail.com (A.T.); cosimo.denunzio@uniroma1.it (C.D.N.); 2 Department of Urology, San Luigi Hospital, Orbassano, 10043 Turin, Italy; cristian_fiori@icloud.com; 3 Department of Urology, Niguarda Hospital, 20162 Milan, Italy; silvia.secco@ospedaleniguarda.it (S.S.); alberto.olivero@ospedaleniguarda.it (A.O.); 4 Department of Urology, Villa Stuart, 00135 Rome, Italy; lucacindolo@virgilio.it

**Keywords:** benign prostatic hyperplasia, minimally invasive surgery, LUTS

## Abstract

**Background:** The aim of this study was to evaluate treatment satisfaction and decision regret in patients undergoing minimally invasive surgical therapies (MISTs) for lower urinary tract symptoms (LUTS) due to benign prostatic hyperplasia (BPH). **Materials and Methods:** We analyzed prospectively collected data from consecutive patients undergoing MISTs across five Italian primary care urology centers. All patients underwent a comprehensive clinical assessment, including detailed medical history and physical examination. Preoperative, perioperative, and postoperative variables were recorded. Decision regret was assessed using validated questionnaires, with significant regret defined as a score >25%. **Results:** A total of 155 patients were included, with a median age of 64 years (IQR 58–66) and a median IPSS of 23 (IQR 18–26). Among them, 90 patients (51%) underwent Aquablation, 21 (12%) received a temporary implantable nitinol device (iTIND), 26 (15%) underwent water vapor thermal therapy (WVTT), and 37 (21%) were treated with prostatic urethral lift (PUL). The overall median decision regret score was 0 (IQR 0–15), with 23 patients (15%) reporting significant regret (>25%). Higher regret rates were observed in patients treated with PUL and WVTT compared to those undergoing iTIND and Aquablation. None of the evaluated variables—including age, BMI, prostate volume, preoperative Qmax, or preoperative IPSS—were significantly associated with treatment regret. However, although not reaching statistical significance, a prostate volume >60 cc was associated with higher regret in patients undergoing WVTT (OR = 3.33) and PUL (OR = 4.2). **Conclusions:** Among patients undergoing MISTs, treatment regret is not negligible and appears higher when patient selection is suboptimal. Larger studies are warranted to better identify predictors of decision regret and optimize patient selection for these procedures.

## 1. Introduction

Lower urinary tract symptoms (LUTS) secondary to benign prostatic hyperplasia (BPH) are a common condition in aging males, significantly impacting quality of life. While transurethral resection of the prostate (TURP) has been considered the gold standard for patients with moderate-to-severe LUTS refractory to medical therapy, minimally invasive surgical techniques (MISTs) have emerged as an alternative treatment options [1,2,3]. These procedures aim to provide symptom relief with fewer perioperative complications, shorter recovery times, and reduced morbidity compared to traditional surgical approaches [4]. Additionally, MISTs have been shown to better preserve ejaculatory function compared to TURP, making them an attractive option for patients concerned about sexual function [5]. Among the available MISTs, Aquablation, prostatic urethral lift (PUL), water vapor thermal therapy (WVTA), and temporary implantable nitinol devices (iTIND) have gained increasing adoption [6,7,8,9,10]. Despite the advantages associated with MISTs, patient-reported outcomes (PROs) and treatment regret remain critical aspects of post-treatment evaluation [11,12].

PROs capture the patient’s perspective on symptom relief, quality of life, and overall satisfaction, allowing for a comprehensive understanding of the impact of treatment beyond clinical measure [13,14,15,16]. Treatment regret reflects discrepancies between patient expectations and actual postoperative experiences, which can significantly influence long-term satisfaction and adherence to treatment recommendations [17,18,19,20]. Factors contributing to treatment regret include symptom persistence, adverse effects, and psychological distress related to unmet expectations. While previous studies have evaluated the efficacy and safety of individual MIST procedures, limited data exist on the comparative treatment regret among different MISTs. For instance, recent analyses have indicated that even if PUL and WVTA provide symptom relief, they may be associated with a higher likelihood of retreatment compared to other MISTs [8]. Complications associated with these procedures, including urinary retention, dysuria, and transient incontinence, may also contribute to dissatisfaction and regret. Additionally, emerging evidence suggests that larger prostate volumes (>60 cc) may be associated with an increased risk of treatment regret, particularly in patients undergoing WVTA and PUL. The role of perioperative factors, such as catheter dependency and urinary function recovery, also plays a key role in post-treatment satisfaction [21]. Understanding the predictors of treatment regret is essential to optimize patient selection, improve shared decision-making processes, and enhance long-term patient satisfaction.

The present study aims to evaluate treatment satisfaction and treatment regret in patients undergoing MISTs for LUTS/BPH across different primary care urology centers, with the objective of identifying potential predictors of regret and informing future treatment strategies.

## 2. Materials and Methods

A consecutive cohort of patients undergoing minimally invasive surgical therapies (MISTs)—including Aquablation, prostatic urethral lift (PUL), water vapor thermal therapy (WVTT), and temporary implantable nitinol devices (iTIND)—for benign prostatic hyperplasia (BPH) was prospectively enrolled across four Italian primary care urology centers between January 2019 and January 2022. Following informed consent, treatment satisfaction and regret were assessed in July 2023.

Inclusion criteria comprised men aged ≥50 years with symptomatic lower urinary tract symptoms (LUTS) due to BPH, refractory to medical therapy and deemed suitable for surgical intervention. Exclusion criteria included a history of prostate cancer, neurogenic bladder dysfunction, follow-up shorter than 18 months, or prior prostate surgery. Patients who failed to complete the questionnaires or provided inadequate responses were excluded from the analysis, as regret assessment was not feasible (*n* = 3).

All patients underwent a comprehensive evaluation, including detailed medical history and physical examination. Preoperative, perioperative, and postoperative data were systematically collected.

All procedures were performed by experienced surgeons who had surpassed the learning curve for both endoscopic surgery and the respective MIST techniques. Follow-up assessments included total PSA measurement, uroflowmetry, post-void residual volume evaluation, and the International Prostate Symptom Score (IPSS). Retreatment rates were also recorded.

Decision regret was evaluated in July 2023 using the Decision Regret Scale (DRS) in patients with a minimum follow-up of 18 months. The DRS is a validated self-administered questionnaire designed to quantify regret related to healthcare decisions [17]. The DRS consists of five items, each rated on a 5-point Likert scale ranging from 1 (“strongly agree”) to 5 (“strongly disagree”). According to standard methodology, negatively worded items (items 2 and 4) were reverse-coded, and the mean score across all five items was calculated. This mean value was then transformed to a 0–100 scale using the formula: (mean score − 1) × 25, where a score of 100 indicates the highest level of regret [17]. A threshold of 25 was used to distinguish between low and high levels of decision regret [22,23,24,25].

### Statistical Analysis

Data are reported as median (interquartile range, IQR) and frequencies. Group differences were assessed using the Mann–Whitney U test for continuous variables and the chi-square test for categorical variables. Decision regret and its associations with relevant predictor variables were systematically analyzed.

Given the skewed distribution of DRS scores, logistic regression was selected as the most appropriate approach to evaluate the relationship between decision regret and potential predictors. Univariable and age-adjusted binary logistic regression analyses were performed to identify factors associated with high regret. Variables with *p* < 0.05 in the univariable analysis were subsequently included in the multivariable model. Odds ratios (ORs) with corresponding 95% confidence intervals (CIs) were calculated.

All statistical analyses were conducted using IBM SPSS Statistics 25.0 (IBM Corp., Armonk, NY, USA).

## 3. Results

A total of 155 patients were enrolled with a median follow-up of 24 months (18–30), with a median age of 64 years (IQR: 58–69) and a median International Prostate Symptom Score (IPSS) of 23 (IQR: 18–27). Among them, 90 patients (58%) underwent aquablation, 11 (7%) received a temporary implanted nitinol device (iTIND), 26 (17%) underwent water vapor thermal ablation (WVTA), and 28 (18%) underwent a prostatic urethral lift (PUL) (Table 1).

The overall median treatment regret score was 0 (IQR: 0–15), with 24 out of 155 patients (17%) experiencing significant regret (DRS > 25). Pre-operative characteristics such as age, prostate volume, BMI, PSA, use of alpha-blockers or 5ARIs, IPSS and QoL scores did not significantly differ between patients presenting regret vs. satisfaction (Table 2). When looking at different MISTs, 6/90 (7%) in the acquablation group, 1/11 (9%) in the iTind group, 8/26 (31%) in the water thermal vapor group and 9/27 (33%) in the prostatic urethral lift group presented surgical regret (*p* < 0.001). Patients with surgical regret presented lower improvements in terms of maximum flow rate (ΔQmax: 0.35 mL/s vs. ΔQmax: 7 mL/s; *p* < 0.05) and IPSS (ΔIPSS: 6 vs. ΔIPSS: 12; *p* < 0.05).

In terms of post-operative functional outcomes, patients undergoing different surgical techniques presented statistically significant improvements in terms of IPSS, Qmax and QoL (Table 3).

On binary logistic regression analysis, improvements in Qmax and IPSS were a predictor of surgical regret. More specifically the risk of regret was 15% lower per ml in Qmax improvement and 7% per IPSS point (Table 4).

## 4. Discussion

To the best of our knowledge, this is one of the few studies that evaluate patient satisfaction and treatment regret following minimally invasive surgical techniques (MISTs) for lower urinary tract symptoms (LUTS) secondary to benign prostatic hyperplasia (BPH). The present study highlights a low risk of treatment regret in patients undergoing iTind and acquablation procedures ranging from 7 to 9% while patients undergoing PUL or WVTA have a significant risk of regret around 30%. According to our results, poor improvements in IPSS and Qmax were significant predictors of treatment regret in our cohort. However, all the techniques showed statistically significant improvements in terms of Qmax and IPSS.

The present study provides important new insights into the management of LUTS associated with BPH. In recent years, minimally invasive surgical therapies (MISTs) have gained increasing popularity; however, outcomes achieved with these novel techniques are frequently suboptimal and not always comparable to those of established surgical procedures [26,27,28]. Our findings clearly demonstrate that the proportion of patients experiencing decision regret is considerable, reaching 17%, a notably high rate for surgery performed for a benign condition.

In our cohort, no preoperative predictors of surgical regret were identified, a result consistent with existing literature, which suggests that surgical regret is primarily driven by postoperative outcomes rather than baseline characteristics [17,18,23,29,30,31]. In line with this concept, postoperative symptom burden and urinary flow parameters were the only significant predictors of regret in our analysis. In our study, we evaluated regret in patients with at least 18 months of follow-up in order to minimize the potential influence of transient postoperative symptoms. A key strength of our study is the assessment of surgical regret after urinary symptoms and flow parameters had stabilized. However, we did not collect data on immediate postoperative regret, which may represent a limitation of the present study.

The lower rate of regret observed among patients undergoing aquablation is plausibly explained by their favorable postoperative outcomes, as consistently reported in the literature [32,33,34]. Conversely, the higher regret rates among patients treated with UroLift are likely attributable to the more modest symptomatic improvements associated with this technique [35].

Overall, the main message of this study is that patients considered for MISTs require careful preoperative evaluation, with particular attention to expectation management, which remains a critical determinant of post-operative satisfaction. In the future, studies should also focus on additional potential determinants of regret, such as patient expectations, the quality of preoperative counseling, and the degree of involvement in shared decision-making [36,37]. As highlighted by previous studies, these factors represent major contributors to surgical regret.

Despite the increasing adoption of MISTs to minimize adverse events, these procedures may present some complications and be suboptimal in terms of symptoms and flow improvements. Such complications inevitably impact patients’ quality of life and influence their level of satisfaction or regret with the chosen treatment. Therefore, a multidisciplinary approach involving urologists and other specialists is essential to provide comprehensive preoperative counseling. More specifically, younger patients with BPH may benefit from the involvement of andrologists and psychologists to better assess individual needs and expectations. However, there is currently no evidence demonstrating that a multidisciplinary approach improves patient-reported outcomes, although dedicated studies are ongoing to address this question. Ensuring that patients are well informed about the efficacy and potential risks associated with different treatment options can reinforce their confidence in the selected procedure, even when adverse effects occur. This study aimed to identify the primary factors associated with satisfaction and regret in patients undergoing MISTs. The Decision Regret Scale (DRS), a validated tool for quantifying regret related to healthcare decisions, was administered to all participants [29]. The importance of analyzing patient satisfaction over time is emphasized by the fact that while early postoperative urinary outcomes can be assessed, long-term functional recovery, including continence and sexual function, may take months to stabilize. Our study adopted a minimum follow-up period of 18 months, ensuring a reliable evaluation of postoperative satisfaction and regret.

Overall, 85% of patients were classified in the low regret group, whereas 15% scored higher than 25 on the DRS questionnaire. Multivariate analysis identified IPSS, Qmax, and the selected MIST as key predictors of regret. These findings contrast with previous studies that did not find significant associations between decision regret and the type of surgical technique used [19,23,30,31,38,39]. One possible explanation is that previous cohorts consisted of highly proactive patients who played an active role in selecting their treatment and surgical setting, which may have mitigated regret. In our study, however, we did not assess patients’ preoperative knowledge regarding MISTs, nor their role in the decision-making process—factors that could significantly influence postoperative satisfaction. Recent studies on decision regret have shown that patient expectations significantly impact postoperative outcomes [23]. Particularly, different MISTs have different functional outcomes, durability and postoperative courses [32,33,40,41,42]. Our study supports these findings, further emphasizing the importance of patient education and expectation management. A strong correlation exists between LUTS improvement and reduced treatment regret. Several reports have demonstrated that persistent urinary symptoms postoperatively are associated with increased regret and lower quality of life. Patient-centered care and careful patient selection are essential when considering minimally invasive surgical therapies (MISTs) in order to minimize the risk of postoperative dissatisfaction or surgical regret. No single MIST is suitable for all patients, and, likewise, individual patients may not be willing to accept the specific characteristics, limitations, or trade-offs associated with a given technique. Therefore, the selection of a MIST should extend beyond a purely anatomical or disease-based assessment. Clinicians must also take into account the patient’s social context, personal values, and expectations, as well as those of their partner, particularly with respect to functional outcomes and quality of life. In this setting, the recent introduction of artificial intelligence and natural language processing models may further enhance patient education and improve patient–physician communication [43,44,45]. By facilitating clearer information exchange and shared decision-making, these tools have the potential to reduce decisional conflict and ultimately lower the risk of patient regret.

Nowadays two other important MISTs are gaining attention: Optilume and transperineal laser ablation (TPLA). Optilume has reached two years of follow-up and results have been published recently [46]. The Optilume BPH Catheter System has received regulatory approval as a minimally invasive surgical therapy for the treatment of lower urinary tract symptoms (LUTS) secondary to benign prostatic hyperplasia (BPH) [46]. Its mechanism of action combines mechanical prostatic urethral dilation with the localized delivery of paclitaxel via a drug-coated balloon, aiming to relieve obstruction while inhibiting restenosis and tissue regrowth, thereby enhancing durability of symptom relief. In the randomized PINNACLE study, 148 men with symptomatic BPH were allocated in a 2:1 ratio to Optilume BPH or sham treatment. Long-term follow-up of up to 2 years was available for patients treated with Optilume [46]. At 24 months, 67.5% of treated patients achieved durable symptomatic response, defined as a ≥30% improvement in IPSS without the need for medical or surgical retreatment. Mean IPSS decreased substantially from 23.4 to 11.0, accompanied by a marked improvement in Qmax (+116.8%) and a modest reduction in post-void residual volume. Improvements in uroflowmetry were consistent across prostate sizes [46]. Quality-of-life outcomes, including BPH Impact Index and IPSS-QoL, demonstrated sustained and clinically meaningful benefits through 2 years. The safety profile was favorable, with mostly transient adverse events such as hematuria and urinary tract infection, no late serious device-related events, and no negative impact on sexual function. Overall, these findings support Optilume BPH as a safe and durable minimally invasive option with outcomes comparable to more invasive therapies while preserving sexual function. However, data on surgical regret is still not available. On the other side, TPLA has shown interesting results for the treatment of BPH [47,48]. Transperineal Laser Ablation of the Prostate (TPLA) has emerged as an ultra-minimally invasive treatment option for lower urinary tract symptoms (LUTS) due to benign prostatic hyperplasia (BPH), designed to overcome the morbidity associated with standard surgical techniques. TPLA is performed under local anesthesia in an outpatient setting and relies on the transperineal insertion of laser fibers into the prostate under ultrasound guidance. The controlled delivery of laser energy induces precise coagulative necrosis of prostatic tissue, leading to progressive volume reduction while sparing periurethral structures and ejaculatory pathways. A systematic review of the available literature identified 17 studies evaluating TPLA outcomes, including two randomized controlled trials comparing TPLA with transurethral resection of the prostate (TURP), as well as prospective and retrospective non-randomized studies. Despite heterogeneity in patient selection and perioperative management, TPLA consistently resulted in significant and durable improvements in urinary symptoms and functional outcomes, with follow-up extending up to 5 years. Importantly, improvements in LUTS were achieved without clinically meaningful impairment of erectile or ejaculatory function. The safety profile of TPLA was favorable, with predominantly low-grade complications (Clavien–Dindo ≤ II) and no major adverse events reported. Overall, current evidence suggests that TPLA represents a promising ejaculation-sparing alternative for selected patients with BPH. However, given the limited number of high-quality comparative studies, further large-scale prospective trials are needed. Additionally, data on surgical regret is needed to define its role in clinical practice.

In a recent mini-review, Elterman et al. introduced the concept of first-line interventional treatment (FIT), proposing it as a novel therapeutic paradigm positioned between pharmacological therapy and conventional surgery. FIT is intended to provide effective symptom relief through minimally invasive interventions characterized by rapid recovery, favorable safety, outpatient feasibility, durability, and preservation of future treatment options, while prioritizing patient-centered outcomes. However, the authors acknowledge that currently available minimally invasive surgical treatments (MISTs) do not fully meet these criteria, highlighting the need for an optimized FIT strategy [49].

Before advocating FIT as a first-line option, it is essential to determine whether patients truly prefer interventional over medical management and to clarify the relative importance of ejaculatory function in decision-making. Malde et al. reported that 47–67% of patients favor non-surgical management or watchful waiting, while only 7% consider sexual function preservation a primary determinant. In the United States, fewer than 2% of men with moderate-to-severe LUTS undergo TURP, and only 1% opt for alternative interventions. These findings underscore the importance of a patient-centered care model that incorporates social context, partner involvement, illness perception, and primary care influence beyond disease severity alone [50].

Despite its conceptual appeal, FIT implementation faces challenges related to cost, accessibility, long-term durability, reimbursement, and retreatment rates. Moreover, current evidence suggests that MISTs provide symptom and flow improvements inferior to conventional surgery. The most widely adopted MISTs—water vapor therapy, UroLift, and iTind—achieve IPSS reductions of 8–12 points and Qmax increases of 2–4 mL/s, outcomes more comparable to pharmacological therapy than to established surgical techniques [51,52].

The integration of artificial intelligence (AI) and natural language processing (NLP) has the potential to support clinicians and patients in selecting the most appropriate surgical strategy for benign prostatic hyperplasia (BPH). AI-driven tools can facilitate shared decision-making by synthesizing clinical variables, guideline recommendations, and patient preferences into personalized, evidence-based suggestions [53,54,55]. NLP systems trained on high-quality datasets can translate complex medical information into clear, patient-friendly language, improving health literacy and engagement while generating tailored educational materials and informed consent documentation, thereby reducing decisional conflict [56,57,58,59,60,61,62,63].

In parallel, large-scale clinical data have enabled predictive analytics and digital twin models—virtual representations of individual patients integrating demographic and clinical data to simulate disease progression and treatment response. In BPH surgery, digital twins may help forecast functional outcomes, complication risk, and satisfaction more accurately than traditional tools, allowing scenario testing across surgical techniques. Although still in early clinical adoption, AI-based decision-support systems hold promise for advancing precision medicine in BPH care. Future validation and regulatory oversight will be essential to ensure reliability and ethical implementation [64].

Despite these valuable insights, our study has several limitations. One major limitation is the lack of baseline quality-of-life data. Another limitation concerns the level of patient knowledge regarding potential side effects. We did not assess whether participants had been adequately informed or the nature of their role in the decision-making process. Given that uninformed patients may have unrealistic expectations, this factor may have influenced their level of regret. Additionally, the voluntary nature of study participation introduces the possibility of nonresponse bias. Patients who chose to participate may not fully represent the broader population undergoing MISTs, potentially limiting the generalizability of our findings. Moreover, the groups were highly unbalanced, which represents a significant limitation and potential source of bias. However, real-world data on patient-reported outcomes following MIST are currently limited; therefore, our study contributes valuable evidence to this underexplored area. Finally, the arbitrary selection of a DRS score of 25 as the cutoff for defining high versus low regret may not fully capture the complexity of the decision-regret phenomenon. While this threshold has been used in prior studies, further research is required to standardize this measure. Another possible limitation is the lack of a multivariable analysis due to the low number of events (24) which only allowed for an age adjusted analysis. Finally, this was an Italian multicenter study, and our findings reflect the characteristics of the enrolled population. Therefore, the results may not be generalizable to cohorts from other countries or to populations with different demographic and clinical profiles.

Despite these limitations, our study provides valuable insights into the predictive factors associated with treatment regret and satisfaction following MISTs. Our findings highlight that while the primary goal remains symptomatic relief, postoperative quality of life is a major determinant of patient satisfaction. In some cases, high regret levels may lead patients to question their treatment choice. To minimize the risk of regret, thorough and collaborative preoperative counseling is essential. Discussions should address surgical expectations, potential complications, and long-term functional outcomes. This is particularly crucial for patients with mild or moderate BPH, for whom alternative treatments may be equally viable. Enhancing patient awareness and ensuring informed decision-making can help align expectations with postoperative realities, ultimately improving patient satisfaction and reducing treatment regret.

## 5. Conclusions

The role of minimally invasive surgical therapies (MISTs) in the management of benign prostatic hyperplasia (BPH) is becoming increasingly prominent. Decision regret in these patients clearly depends on surgical outcomes. Patients’ expectations are clearly essential particularly when choosing a MIST which may not completely solve symptoms and flow when compared to classic techniques. The present study claims particular attention to patient selection and expectations when choosing a MIST in a patient with LUTS and BPH.

## Figures and Tables

**Table 1:** Overall characteristics and regret. Abbreviations: iTIND = temporary implanted nitinol device; WVTA = water vapor thermal ablation; PUL= prostatic urethral lift.

Total = 155		Median (IQR)/n (%)
Age		64 (58–69)
TRUS volume		58 (45–70)
BMI		25.6 (23.9–27.4)
PSA		2.5 (1.2–4.0)
Alfa-blockers		127/155 (82%)
5ARI		16/155 (10%)
Indwelling CV		11/155 (7%)
IPSS		23 (18–27)
Nocturia episodes		4 (2–5)
QoL		4 (4–5)
Pre-operative Qmax		8.0 (6.9–10.1)
Technique		
Acquablation		90/155 (58%)
iTIND		11/155 (7%)
WVTA		26/155 (17%)
PUL		28/155 (18%)
Surgical Time		60 (53–73)
Complications		
1		8/155 (5%)
2		4/155 (3%)
3		1/155 (1%)
4		0/155 (0%)
5		0/155 (0%)
Catheterization time		3 (3–3)
Post-operative Qmax		15.0 (11.4–20.0)
PVR		20 (1–40)
REGRET		0 (0–15)
REGRET	≤25	131/155 (83%)
	>25	24/155 (17%)
REGRET by technique		
Acquablation		6/90 (7%)
iTIND		1/11 (9%)
WVTA		8/26 (31%)
PUL		9/28 (32%)

**Table 2:** Patients with and without regret: Univariate analysis.

Total = 155	No Regret (131/155) (83%) Median (IQR) n (%)	Regret (24/155) (17%) Median (IQR) n (%)	*p*
Age	64 (58–69)	65 (55–71)	0.57
TRUS volume	58 (45–70)	58 (41–75)	0.97
BMI	26 (24–27)	26 (23–29)	0.79
PSA	2.5 (1.2–3.9)	2.4 (1.1–4.0)	0.72
Alfa-blockers	109 (83%)	18 (75%)	0.39
5ARI	12 (9%)	4 (17%)	0.78
Indwelling CV	9 (7%)	2 (8%)	0.68
IPSS	23 (18–27)	23 (16–24)	0.53
Nocturia episodes	4 (2–5)	4 (2–5)	0.81
QoL	5 (4–5)	4 (4–5)	0.30
Pre-operative Qmax	8 (7–10)	9 (7–12)	0.29
Technique			<0.001
Acquablation	84 (64%)	6 (25%)	
iTIND	10 (8%)	1 (4%)	
WVTA	18 (14%)	8 (33%)	
PUL	19 (14%)	9 (38%)	
Surgical Time	60 (54–72)	66 (51–80)	0.67
Complications			0.87
1	7 (5%)	0	
2	4 (3%)	1 (4%)	
3	1 (0.8%)	0	
4	0	0	
5	0	0	
Catheterization time	3 (3–3)	3 (3–9)	0.60
Post-operative IPSS at follow-up	11 (8–15)	15 (12–20)	0.34
ΔIPSS	12 (10–12)	6 (4–10)	0.01
Post-operative Qmax at follow up	15 (12–20)	10 (7–14)	<0.001
ΔQmax	7 (3.2–11.2)	0.35 (−0.13–3.7)	<0.001

**Table 3:** Pre- and postoperative surgical outcomes.

	Pre-Operative	Post-Operative at Follow-Up	*p*
Qmax	8.0 (6.9–10.1)	15.0 (11.4–20.0)	<0.001
Acquablation	8.0 (6.8/10)	17 (13/22)	<0.001
iTIND	8.7 (8.0/11.0)	11 (9.5/12,5)	<0.001
WVTA	8.0 (6.7/11.0)	12.6 (9.2/18)	<0.001
PUL	8.8 (6.5/13.6)	12.2 (9.5/14.8)	<0.001
IPSS	23 (18/27)	11 (7/18)	<0.001
Acquablation	22 (16/25)	12 (8/18)	<0.001
iTIND	17 (16/23)	10 (7/5)	<0.001
WVTA	22 (16/25)	15 (10/18)	<0.001
PUL	16 (13/20)	12 (7/18)	<0.001
QoL	4 (4/5)	1 (1/2)	<0.001
Acquablation	4 (4/5)	2 (1/2)	<0.001
iTIND	5(4/5)	1 (1/2)	<0.001
WVTA	4 (4/5)	2 (1/2)	<0.001
PUL	5(5/5)	1 (1/2)	<0.001

**Table 4:** Binary logistic regression for the risk of regret.

Variable	OR	*p*	Age Adjusted OR	*p*
Age	1.01 (0.96–1.07)	0.63	--	-
TRUS volume	0.99 (0.97/1.01)	0.91	0.99 (0.97/1.03)	0.93
BMI	1.02 (0.85/1.23)	0.79	1.04 (0.80/1.43)	0.65
PSA	0.99 (0.82–1.19)	0.92	0.99 (0.80–1.19)	0.89
Indwelling CV	1.23 (0.25/6.09)	0.80	1.56 (0.56/7.19)	0.77
IPSS	0.97 (0.90–1.05)	0.45	0.98 (0.89–1.09)	0.35
Nocturia episodes	0.93 (0.53–1.62)	0.80	0.90 (0.50–1.52)	0.78
QoL	0.79 (0.49–1.27)	0.32	0.70 (0.30–1.47)	0.32
Pre-operative Qmax	1.08 (0.95–1.22)	0.82	1.09 (0,93–1.27)	0.89
Surgical Time	1.01 (0.95–1.07)	0.81	1.07 (0.90–1.10)	0.84
Post-operative IPSS	1.08 (1.02–1.18)	0.020	1.09 (1.03–1.20)	0.037
ΔIPSS	1.07 (1.01–1.20)	0.003	1.07 (1.01–1.20)	0.008
Post-operative Qmax	0.80 (0.70–0.91)	<0.001	0.86 (0.71–0.96)	<0.001
ΔQmax	0.85 (0.77–0.95)	<0.001	0.80 (0.77–0.97)	<0.001
Acquablation	0.19 (0.07–0.50)	<0.001	0.17 (0.05–0.50)	<0.001
iTind	0.52 (0.06–4.31)	0.550	0.62 (0.06–4.51)	0.560
WVTA	3.31 (1.17–8.39)	0.023	3.41 (1.29–9.39)	0.033
Urolift	3.53 (1.35–9.22)	0.010	3.76 (1.35–9.46)	0.001

## Data Availability

The original contributions presented in this study are included in the article. Further inquiries can be directed to the corresponding author.
